# Comparative Analysis of Microfluidics Thrombus Formation in Multiple Genetically Modified Mice: Link to Thrombosis and Hemostasis

**DOI:** 10.3389/fcvm.2019.00099

**Published:** 2019-07-30

**Authors:** Magdolna Nagy, Johanna P. van Geffen, David Stegner, David J. Adams, Attila Braun, Susanne M. de Witt, Margitta Elvers, Mitchell J. Geer, Marijke J. E. Kuijpers, Karl Kunzelmann, Jun Mori, Cécile Oury, Joachim Pircher, Irina Pleines, Alastair W. Poole, Yotis A. Senis, Remco Verdoold, Christian Weber, Bernhard Nieswandt, Johan W. M. Heemskerk, Constance C. F. M. J. Baaten

**Affiliations:** ^1^Department of Biochemistry, Cardiovascular Research Institute Maastricht (CARIM), Maastricht University, Maastricht, Netherlands; ^2^Rudolf Virchow Center, Institute of Experimental Biomedicine, University Hospital Würzburg, University of Würzburg, Würzburg, Germany; ^3^Wellcome Trust Sanger Institute, Cambridge, United Kingdom; ^4^Department of Vascular Surgery, Experimental Vascular Medicine, Heinrich Heine University, Düsseldorf, Germany; ^5^Institute of Cardiovascular Sciences, College of Medical and Dental Sciences, University of Birmingham, Birmingham, United Kingdom; ^6^Institute of Physiology, University of Regensburg, Regensburg, Germany; ^7^GIGA-Cardiovascular Sciences, University of Liège, Liège, Belgium; ^8^Medizinische Klinik und Poliklinik I, Klinikum der Universität München, Ludwig-Maximilians-University, and DZHK (German Center for Cardiovascular Research), Partner Site Munich Heart Alliance, Munich, Germany; ^9^Department of Physiology and Pharmacology, School of Medical Sciences, University of Bristol, Bristol, United Kingdom; ^10^Institute for Cardiovascular Prevention (IPEK), Ludwig-Maximilians-Universität München, Munich, Germany; ^11^Institute for Molecular Cardiovascular Research (IMCAR), University Hospital Aachen, Aachen, Germany

**Keywords:** arterial thrombus formation, bleeding, collagen, glycoprotein VI, platelets, microfluidics

## Abstract

Genetically modified mice are indispensable for establishing the roles of platelets in arterial thrombosis and hemostasis. Microfluidics assays using anticoagulated whole blood are commonly used as integrative proxy tests for platelet function in mice. In the present study, we quantified the changes in collagen-dependent thrombus formation for 38 different strains of (genetically) modified mice, all measured with the same microfluidics chamber. The mice included were deficient in platelet receptors, protein kinases or phosphatases, small GTPases or other signaling or scaffold proteins. By standardized re-analysis of high-resolution microscopic images, detailed information was obtained on altered platelet adhesion, aggregation and/or activation. For a subset of 11 mouse strains, these platelet functions were further evaluated in rhodocytin- and laminin-dependent thrombus formation, thus allowing a comparison of glycoprotein VI (GPVI), C-type lectin-like receptor 2 (CLEC2) and integrin α_6_β_1_ pathways. High homogeneity was found between wild-type mice datasets concerning adhesion and aggregation parameters. Quantitative comparison for the 38 modified mouse strains resulted in a matrix visualizing the impact of the respective (genetic) deficiency on thrombus formation with detailed insight into the type and extent of altered thrombus signatures. Network analysis revealed strong clusters of genes involved in GPVI signaling and Ca^2+^ homeostasis. The majority of mice demonstrating an antithrombotic phenotype *in vivo* displayed with a larger or smaller reduction in multi-parameter analysis of collagen-dependent thrombus formation *in vitro*. Remarkably, in only approximately half of the mouse strains that displayed reduced arterial thrombosis *in vivo*, this was accompanied by impaired hemostasis. This was also reflected by comparing *in vitro* thrombus formation (by microfluidics) with alterations in *in vivo* bleeding time. In conclusion, the presently developed multi-parameter analysis of thrombus formation using microfluidics can be used to: (i) determine the severity of platelet abnormalities; (ii) distinguish between altered platelet adhesion, aggregation and activation; and (iii) elucidate both collagen and non-collagen dependent alterations of thrombus formation. This approach may thereby aid in the better understanding and better assessment of genetic variation that affect *in vivo* arterial thrombosis and hemostasis.

## Introduction

Current concepts of platelet activation pathways in thrombosis and hemostasis rely to a large extent on the summation of single observations. Frequently, the role of a particular protein or signaling pathway is deduced from the consequences of a genetic knockout in mouse on platelet responses, such as in comparison to changes in experimental arterial thrombosis and tail bleeding. A large set of such studies has resulted in the concept of collagen-induced arterial thrombus formation (1–4). Herein, it is stipulated that the exposure of subendothelial collagen to flowing blood is a key trigger to start shear-dependent thrombus formation. Collagen causes platelet adhesion and furthermore binds von Willebrand factor (VWF), which can decelerate flowing platelets at high shear rate. Firm VWF/collagen-mediated adhesion and subsequent platelet activation requires synergy between the VWF receptor, glycoprotein (GP)Ib-V-IX, and the collagen receptors, GPVI and integrin α_2_β_1_ (5–8).

Collagen is known to induce a number of stimulating pathways via GPVI, in particular: (i) activation of Src-family and Syk tyrosine kinases, resulting in phospholipase C (PLC)γ2 activation and intracellular Ca^2+^ mobilization (2, 9); (ii) additional Ca^2+^ influx via ORAI1 channels which couple to the calcium sensor STIM1 in the reticular membrane (10); (iii) activation of several isoforms of protein kinase C (PKC) (11), phosphoinositide 3-kinases (PI3K) (12), and small GTPases, the latter including CDC42, RAC1 and RHOA (13). Additional, modifying signaling pathways include: (iv) activation of phospholipase D, augmenting flow-dependent platelet activation (14); and (v) activation of multiple (tyrosine) phosphatases, a part of which act downstream of immunoreceptor tyrosine-based inhibition motif (ITIM)-containing receptors. Such phosphatases can have a direct or indirect platelet-stimulating (e.g., CD148, DUSP) or a platelet-inhibiting effect (PECAM1, G6b-B) in response to collagen (15). Together, these signaling routes co-operate to control the activation state of the platelet fibrinogen receptor, integrin α_IIb_β_3_, the secretion of granular contents, and the release of thromboxane-A_2_. Locally secreted or generated soluble agonists, acting via G-protein coupled receptors (GPCR) ensure the capture and incorporation of passing platelets into an aggregate or thrombus (2–4). In a subset of platelets in the thrombus, procoagulant activity is generated by Ca^2+^-dependent activation of the anoctamin-6 (TMEM16F) channel (16), regulating phosphatidylserine (PS) exposure and platelet ballooning (17–19).

Although all these signaling components are known to play a certain role in collagen-dependent thrombus formation, there is still limited insight into the relative contribution of individual proteins. In addition, it is unclear to which extent C-type lectin-like receptor-2 (CLEC2), another receptor that signals via tyrosine kinases (20, 21), is capable to regulate the process of thrombus formation. The same holds for integrin α_6_β_1_, an adhesive receptor, which mediates flow-dependent adhesion of platelets to the matrix protein laminin (22).

In a recent synthesis approach, a quantitative evaluation was made of the contribution of 431 mouse genes to experimental arterial thrombosis and hemostasis *in vivo*, thereby revealing several genes with a role in thrombosis without affecting bleeding (23). For the total cohort of studies and mouse genes, it appeared that microfluidics assays where thrombus formation is measured *in vitro*—by whole blood perfusion over a collagen surface—predict the consequences of a gene knockout on thrombosis models *in vivo* (23). However, the standard microfluidic tests only report on changes in platelet adhesion (surface area coverage, SAC%), which is a limitation given that the recorded microscopic images also contain information on platelet aggregate formation (24, 25). In comparison, for human blood samples from a large cohort of healthy subjects, it could be shown that a multi-parameter image analysis can provide detailed information on the sub-processes of platelet adhesion, aggregation and activation at the same time (26).

To better understand the alterations in thrombus phenotypes using microfluidics, we applied a similar multi-parameter approach to quantitatively compare the effects of deficiency of 37 signaling proteins on collagen-induced pathways. We therefore re-analyzed earlier recorded microscopic images, in all cases from thrombi generated using the same microfluidic flow chamber setup.

## Materials and Methods

### Mice

Mice were included from 38 strains, in each case with a monogenetic or antibody-induced deficiency, as well as 22 sets of corresponding wild-type or control mice, as described in the original publications (see Table 1). As a selection criterion for inclusion, microscopic images needed to be available from *n* ≥ 3 animals per modified group and *n* ≥ 4 for wild-types. Scaled effects on *in vivo* arterial thrombosis and tail bleeding are for the majority of strains described previously (23).

**Table 1:** Overview of re-analyzed data sets on thrombus formation using the Maastricht flow chamber.

**Gene**	**Protein**	**Genetic modification**	**Background**	**DB**	**Δ** **SAC%**	**Thrombosis phenotype**	**Bleeding phenotype**	**Remarks**	**References**
*Ano1*	Anoctamin-1	PF4-Cre *Ano*1	C57Bl/6	07	o	n.d.	n.d.	-	(18)
*Ano6*	Anoctamin-6	*Ano6*^Gt(AW0382)^	C57Bl/6	07	o	n.d.	↑	Prolonged bleeding	(18)
*Anxa1*	Annexin A1	PF4-Cre *Anxa*1	C57Bl/6	22	o	n.d.	n.d.	-	(23)^***^
*Apoe*	Apolipoprotein E	*Apoe*^tm1Unc^	C57Bl/6	22	↑	n.d.	n.d.	-	(23)^***^
*Bnip2*	BCL2-interacting protein 2	*Bnip2*^tm1a^	C57Bl/6N	21	o	n.d.	n.d.	-	(23)^***^
*Capn1*	Calpain-1	*Capn1*^tm1Ahc^	C57Bl/6	10	↑	↓	n.d.	Delayed vessel occlusion	(27, 28)
*Cd36*	CD36	*Cd36*^tm1Mfe^	C57Bl/6	14	o	↓	o	Delayed thrombus formation, increased embolization	(29, 30)
*Cdc42*	Small GTPase CDC42	PF4-Cre *Cdc42*	C57Bl/6 x 129SV	20	o	↑	↑	Accelerated vessel occlusion, prolonged bleeding	(31)
*Clec1b*	CLEC2	INU1 Ab^*^	C57Bl/6	19	↓	↓	↑	Delayed thrombus formation, increased embolization	(20)
*Csk*	Tyrosine kinase CSK	PF4-Cre *Csk*_tm1Tara_	C57Bl/6	23	↓	↓	↑	Moderately reduced thrombus formation	(32)
*Dlg4*	Scaffold protein DLG4	*Dlg4*^tm1a^	C57Bl/6N	21	o	n.d.	n.d.	-	(23)^***^
*Dusp3*	Protein phosphatase DUSP3	*Dusp3*^tm1Srah^	C57Bl/6	11	↓	↓	o	Decreased pulmonary embolism, small thrombus volume	(33)
*Fcer1g*	FcR γ-chain	*Fcer1g*^tm1Rav^	C57Bl/6 x 129SV	04	↓	↓	n.d.	Delayed and reduced thrombus formation	(34, 35)
*Fpr2*	Formyl peptide receptor-2	*Fpr2*^Tg(ACTB)#Jimw^	C57Bl/6	22	↑	n.d.	n.d.	-	(23)^***^
*Gnaq*	Gq α-subunit	*Gnaq*^tm1Soff^	C57Bl/6 x 129SV	04	↓	↓	↑	Intra-abdominal bleeding, frequent postnatal death, protection thromboembolism	(34, 36)
*Gp6*	GPVI	*Gp6*^tm1Beni^	C57Bl/6	03	↓	↓	o	Reduced thrombus stability, enhanced embolization	(37, 38)
*Gp6 / Clec1b*	GPVI/CLEC2	JAQ1+INU1 Ab^*^	C57Bl/6	03	n.d	↓	↑	Delayed thrombus formation, smaller thrombus volume	(38)^**^
*Grm8*	Glutamate metabotropic receptor 8	*Grm8*^tm1a^	C57Bl/6N	21	o	n.d.	n.d.	-	(23)^***^
*Ifnar1*	Interferon receptor 1	*Ifnar1*^tm1a^	C57Bl/6N	21	↓	n.d.	n.d.	-	(23)^***^
*Itga2*	Integrin α2	LoxP-Cre *Itga2*	C57Bl/6 x 129SV	05	↓	↓	o	Reduced thrombus formation, increased embolization	(5, 39)
*Itgb1*	Integrin β1	Mx-Cre *Itgb1*	C57Bl/6 x 129SV	04	↓	o	o	Unchanged thrombus formation	(5, 34, 40)
*Kcnn4*	K-activated Ca channel-4	*Kcnn4*^tm1Rklr^	C57Bl/6 x 129SV	16	o	n.d.	n.d.	-	(18)
*Mpig6b*	Receptor G6B-b	*Mpig6b*^tm1.1Arte^	C57Bl/6	24	↓	n.d.	↑	Prolonged bleeding	(41)
*Orai1*	Calcium channel ORAI1	BMC *Orai1*^−/−^	C57Bl/6	01	↓	↓	o/↑	Reduced thrombus formation and stability	(42, 43)
*Pik3cg*	PI 3-kinase γ	*Pik3cg*^tm1Wym^	129SV	13	↓	↓	o	Protected from thromboembolic vascular occlusion	(44, 45)
*Plcg2* (GOF)	Phospholipase C-γ2 (GOF)	*Plcg2^*Ali*5^*	C3HeB/FeJ	12	↑	↑	n.d.	Increased pulmonary thromboembolism	(46)
*Pld1*	Phospholipase D1	*Pld1*^tm3Mafr^	C57Bl/6	02	o	↓	o	Reduced thromboembolism, reduced thrombus stability	(14)
*Prkca*	Protein kinase C-α	*Prkca*^Myh6/tetO/1Jmk^	C57Bl/6	06	↓	↓	o	Delayed thrombus formation, no fecal occult blood	(47, 48)
*Prkcd*	Protein kinase C-δ	*Prkcd*^tm1Kin^	C57Bl/6	06	↑	o	n.d.	Unchanged thrombus formation	(47, 49)
*Prkcq*	Protein kinase C-θ	*Prkcq*^tm1Litt^	C57Bl/6	06	↑	n.d.	n.d.	Limited occlusion, thrombus instability	(47)
*Prkd2*	Protein kinase D2	*Prkd2*^tm1.1Daca^	C57Bl/6	17	↓	n.d.	o	Unchanged bleeding	(50)
*Ptprj*	Phosphatase CD148	PF4-Cre *Ptprj*^tm1.1Weis^	C57Bl/6	23	↓	↓	o	Severely compromised thrombus formation	(32)
*Rac1*	Small GTPase RAC1	Mx-Cre *Rac1*	C57Bl/6	08	↓	↓	↑	Reduced thrombus formation and volume, variable but prolonged bleeding	(51)
*Rhoa*	Small GTPase RHO-A	PF4-Cre *Rhoa*	C57Bl/6 x 129SV	08	o	↓	↑	Unstable thrombus formation, increased embolization	(13)^**^
*Stim1*	Regulator STIM1	BMC *Stim1^−^*^/−^	C57Bl/6	01	↓	↓	↑	Reduced thrombus stability, no vessel occlusion	(42, 52)
*Stim2*	Regulator STIM2	*Stim2*^tm1Beni^	C57Bl/6	01	o	n.d.	n.d.	-	(42)
*Syk*	Tyrosine kinase SYK	PF4-Cre *Syk*^tm1(syk)Spwa^	C57Bl/6	09	n.d.	↓	↑	Blood-filled lymphatics, impaired thrombus formation	(53)^**^
*Vps13a*	Vacuolar sorting protein VPS13A	*Vps13a*^tm1a^	C57Bl/6N	21	↓	n.d.	n.d.	-	(23)^***^

*Shown are published changes in platelet deposition (SAC%) on collagen for mice with indicated genetic deficiencies in comparison to wild-type mice. Where indicated, GPVI and/or CLEC2 were rendered non-functional by injection of antibodies. Further indicated are published effects of the gene deficiency (same mouse strain) on in vivo arterial thrombosis, pulmonary thromboembolism and/or (tail) bleeding*.

*BMC, bone-marrow chimera; DB, database; GOF, gain-of-function mutation; n.d., not determined*.

* Antibody-mediated deficiency of CLEC2 and/or GPVI;

** microfluidics data not included in reference;

*** only data for M1 published; *↑ increased/prolonged; ↓ decreased/shortened; o unchanged*.

In all cases, mouse blood was collected into anticoagulant medium, consisting of PPACK (40 μM), unfractionated heparin (5 U/ml) and low molecular weight heparin (fragmin, 50 U/ml). Samples were immediately processed. For original data, experiments were approved by the district government of Lower Franconia (Bezirksregierung Unterfranken) and by the Animal Experimental Committee in Hinxton/Cambridge. For previously published data, experiments were approved by the local Animal Experimental Committees as indicated in the original publications (see references in Table 1).

### Whole Blood Thrombus Formation Under Flow

For all data sets, thrombus formation in whole blood under flow was assessed with the Maastricht flow chamber (depth 50 μm, width 3 mm, length 30 mm) (24). In short, PPACK/heparin anticoagulated blood (400–500 μl) was perfused for 3.5–4.0 min at room temperature at a wall shear rate of 1000 s^−1^ (where indicated 1700 s^−1^) over (i) Horm-type collagen (100 μg/ml, Nycomed Pharma, Munich, Germany), allowing platelet interaction via GPIb, GPVI and integrin α_2_β_1_.

Where specified, two additional surfaces were used, similarly as described before (24): (ii) VWF-binding peptide (VWF-BP, 12.5 μg/ml, obtained from Prof. Dr. R. Farndale, Cambridge University, UK) + laminin (50 μg/ml, from human plasma, Sigma-Aldrich, St. Louis MO, USA; binding integrin α_6_β_1_) (22), and (iii) VWF-BP + laminin + rhodocytin (250 μg/ml, activating CLEC2) (54). Rhodocytin purified from *Calloselasma rhodostoma* (55), was a kind gift of Prof. Dr. K. Clemetson (Bern University, Switzerland).

After blood perfusion, platelet thrombi were rinsed with modified Tyrode's Hepes buffer (pH 7.45, 5 mM Hepes, 136 mM NaCl, 2.7 mM KCl, 0.42 mM NaH_2_PO_4_, 1 mg/ml glucose, 1 mg/ml bovine serum albumin, 2 mM CaCl_2_, 2 mM MgCl_2_ and 1 U/ml heparin), and then stained during 1.5 min flow with one to three fluorescently labeled platelet activation markers. These were: Alexa Fluor (AF)647 (or FITC) conjugated annexin A5 (AF647: 1:200, Invitrogen Life Technologies, Carlsbad, CA, USA/FITC: 1:1000, PharmaTarget, Maastricht, The Netherlands); FITC anti-mouse CD62P mAb (1:40, rat-anti-mouse, Emfret Analytics, Würzburg, Germany); and phycoerythrin (PE)-labeled JON/A mAb (1:20, Emfret Analytics); all diluted in modified Tyrode's Hepes buffer. Residual labels were removed by another perfusion for 2 min with label-free Tyrode's Hepes buffer. Multiple brightfield and (if applicable) fluorescence microscopic images were captured per surface, of which three representative images were re-analyzed in a systematic way (26).

For image recording, different fluorescence microscopic systems were used (see references in Table 1), but always containing a 60/63x oil objective and a sensitive CCD camera for capturing enhanced-contrast, brightfield images.

### Microscopic Image Analysis

For all mouse strains, the recorded 16-bit or 8-bit brightfield and fluorescence images were re-analyzed using the same newly developed scripts (one per image type), written in Fiji (56). Scripts always opened a series of images one-by-one with a loop. In each loop run, background illumination was corrected using a fast Fourier transform bandpass filter, followed by manual setting of a threshold and measurement of the surface area coverage. For brightfield images, a series of Gray morphology conversions was applied to reduce striping and to improve the detection quality. Image conversion steps were as follows: a diamond large sized close, followed by a medium sized circle close and a small circle shaped dilate. Note that the first step increased the pixels that were stronger in regions with many neighboring pixels, the second step then rounded the shapes and additionally reduced straight lines, while the final step (allowing alteration by user-interface) served to obtain the best match with the original image. The Fourier transform filter served to flatten the background areas sufficiently for good analysis, with a minimal impact on the structures. For brightfield images, large structures were filtered down to 60 pixels, for the fluorescence images of annexin A5, integrin and P-selectin images large structures were filtered down to 65 pixels. Small structures were not filtered down, as these contained structures of interest within the adhered platelets (Supplementary Figure 1).

Using these scripts and by visually scoring the unprocessed brightfield images, the following parameters of thrombus formation were obtained (Table 2): surface area coverage of adhered platelets (*P1*, %SAC); platelet aggregate coverage (*P2*, %SAC); thrombus morphology score (*P3*, scale 0-5); thrombus multilayer score (*P4*, scale 0-3); and thrombus contraction score (*P5*, scale 0-3). Scoring was performed in comparison to a set of pre-defined standard images (Supplementary Figure 1). For the assessment of platelet activation, fluorescence images were analyzed for PS exposure (*P6*, %SAC), P-selectin expression (*P7*, %SAC) and integrin α_IIb_β_3_ activation (*P8*, %SAC) (24).

**Table 2:** Overview of microspotted surfaces (*M*) and parameters of image analysis (*P*) for the assessment of thrombus formation.

**M**	**Microspot surface**		**Platelet receptors involved**	
M1	Collagen type I (VWF)^*^		GPIb, GPVI, α_2_β_1_	
M2	Rhodocytin + laminin + VWF-BP		GPIb, CLEC2, α_6_β_1_	
M3	Laminin + VWF-BP		GPIb, α_6_β_1_	
**P**	**Parameter of analysis**	**Image**	**Range**	**Scaling**
**Platelet adhesion**				
P1	Platelet surface area coverage (%SAC)	BF^**^	0 – 93.67	0 – 10
**Thrombus signature**				
P2	Platelet aggregate coverage (%SAC)	BF	0 – 49.17	0 – 10
P3	Thrombus morphology score	BF	0 – 5.0	0 – 10
P4	Thrombus multilayer score	BF	0 – 3.0	0 – 10
P5	Thrombus contraction score	BF	0 – 3.0	0 – 10
**Platelet activation**				
P6	PS exposure (%SAC)	FL	0 – 12.23	0 – 10
P7	P-selectin expression (%SAC)	FL	0 – 29.70	0 – 10
P8	Integrin α_IIb_β_3_ activation (%SAC)	FL	0 – 28.25	0 – 10

* *Binds VWF from plasma*.

** *BF, brightfield; FL, fluorescence*.

Regardless of the image type, parameter values from three images per experiment were averaged, thus resulting in a single value per parameter and experiment. The image analysis and scoring parameters were verified by different observers, who were blinded to the experimental condition. Parameters of experiments from the same mouse strain were combined as proxy measures of platelet adhesion (*P1*), thrombus signature (*P2-5*), and platelet activation (*P6-8*), as described before for human platelets (26).

### Network Analysis

A network of protein-protein interactions was built based on 37 investigated genes, using the STRING database (57), taking into consideration the following settings: 1st shell interactors: <20 interactors, 2nd shell interactors: <60 interactors, confidence level: medium to high. Networks were visualized in Cytoscape version 3.7.0 (58).

Network clustering analysis was performed with the Cytoscape app, MCODE to identify highly interacting nodes using the following settings: degree cutoff: 2, node score cutoff: 0.2, K-core: 2 and maximum depth: 100 (59, 60).

### Data Processing and Statistical Analysis

For each genetically modified strain and corresponding wild-types, image data were averaged to obtain one parameter per surface, of which mean and SD values were calculated. For heatmap representation, mean values were univariate scaled from 0 to 10 per parameter (24). Gene effect heatmaps were constructed by subtracting scaled average values of the wild-type (control) strain from those of the modified strain. For statistical evaluation, a filter was applied, considering changes outside the range of composite mean ± SD as a relevant difference between modification and wild-type (26). Heatmap data were visualized by (unsupervised hierarchical) cluster analysis using the program R (61). For comparison of raw parameter values, a Kendall's tau-b correlation analysis was performed using SPSS (IBM SPSS version 24, Armonk, NY, USA).

## Results

### Microfluidics Analysis of Thrombus Formation on Collagen of Multiple Wild-Type Mouse Datasets

Microscopic brightfield and (annexin A5) fluorescence images were collected from earlier performed whole blood perfusion experiments with blood from 38 different strains of modified mice and 22 corresponding wild-types (Table 1). Included were experiments with strains (in majority published), that were judged to be of sufficiently high quality and power to allow re-analysis by newly developed image analysis scripts (Supplementary Figure 1). From each experiment, five parameters of thrombus formation were obtained (Table 2). Platelet adhesion was quantified by the conventional analysis of platelet SAC% (*P1*). Thrombus signature (26) was composed of four parameters related to the thrombus buildup (Σ*P2-5*), i.e., platelet aggregate SAC% (*P2*), thrombus morphology score (*P3*), thrombus multilayer score (*P4*), and thrombus contraction score (*P5*). As far as available, platelet activation was assessed from fluorescence images of PS exposure (*P6*) (see Supplementary Figure 1).

**Figure 1:**
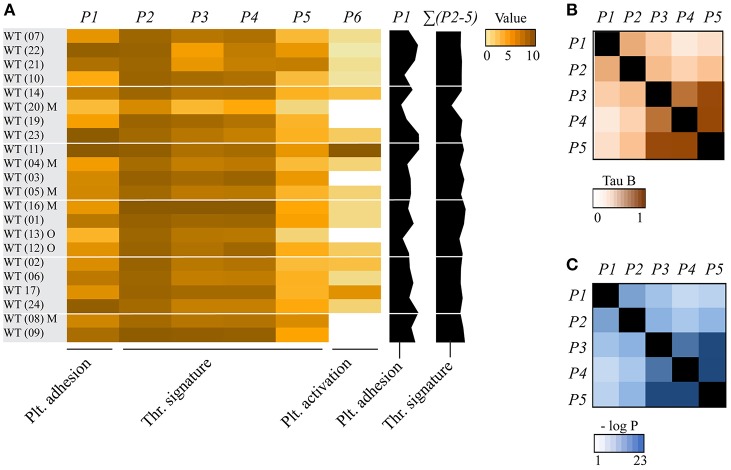
Consistency of collagen-dependent thrombus formation between multiple wild-type mouse datasets. Blood from wild-type (WT) mice (databases as indicated in brackets) was perfused over collagen at a shear rate of 1000 s^−1^ (in two cases 1700 s^−1^), and parameters of thrombus formation were obtained by re-analysis of random brightfield images. Investigated wild-type mice (*n* = 22) had a C57Bl6 genetic background or, where indicated, a mixed C57Bl6 x 129SV background (M) or other background (O). For full details, see Table 1. **(A)** Heatmap of mean parameters, univariate scaled (0–10) across all mouse strains. Parameter clustering was as follows: *platelet adhesion*: *P1* (platelet SAC%); *thrombus signature: P2* (platelet aggregate SAC%), *P3* (thrombus morphology score), *P4* (thrombus multilayer score), *P5* (thrombus contraction score); and *platelet activation*: *P6* (PS exposure). Also indicated (black bars on the right) are the overall scaled values of platelet adhesion (*P1*) and thrombus signature (Σ*P2-5*). The wild-type datasets were arranged based on the alphabetical order of the (genetically) modified mice. **(B,C)** Correlations between parameters of thrombus formation for all cohorts of mice strains. Shown are Kendall's tau-b correlation coefficients **(B)** and corresponding *p*-values **(C)**.

To establish the overall consistency of the combined databases of the whole blood experiments, we compared the mean values plus variation for each of the image analysis parameters *P1-5* for the 22 wild-type control datasets. The calculated coefficients of variation of means across the wild-type datasets ranged from <12% (*P3-5*) to 23-25% (*P1,2*) (Supplementary Table 1). For heatmap presentation, mean values for all wild-types (and corresponding transgenic animals) were univariate scaled from 0-10 (Figure 1A). The obtained heatmap illustrated an overall high cohesion of the data, yet also suggesting that wild-type strains with mixed C57Bl/6 x 129SV background (databases 04, 05, 08, 16, 20) had a tendency for smaller *P3-5* values. Overall, these findings point to a high degree of comparability between the various wild-type datasets.

To determine how the different parameters of collagen-mediated thrombus formation correlated within our dataset of wild-type and genetically modified mice, a correlation matrix was constructed based on the Kendall's tau-b correlation analysis (Figure 1). All parameters showed a significant moderate to strong correlation to each other (Kendall's tau-b: 0.424–0.829). Platelet adhesion (*P1*) correlated strongly to platelet aggregation (*P2*), while it correlated moderately to thrombus morphology (*P3*), multilayer (*P4*) and contraction (*P5*) (Kendall's tau-b: 0.424-0.572). Strongest associations were observed between platelet aggregation (*P2*) and the thrombus scores (*P3-5*) (Kendall's tau-b 0.55–0.65, *p* < 0.001). Furthermore, even stronger correlations were seen between *P3-5*, i.e., the thrombus morphology, multilayer and contraction scores (Kendall's tau-b 0.76–0.83, *p* < 10^−10^).

### Comparing Thrombus Formation on Collagen in Multiple Genetically Modified Mice

As detailed in Table 1, the included 38 modified mouse strains concern animals with defects of single genes, encoding for proteins implicated in GPVI- and/or GPCR-related platelet activation pathways, i.e*., Csk, Fcer1g* (FcR γ-chain), *Gnaq* (Gα_q_), *Orai1, Pik3cg* (PI3Kγ), *Pld1* (PLD1), *Prkca* (PKCα), *Prkcd* (PKCδ), *Prkcq* (PKCθ), *Prkd2* (PKD2), *Stim1, Stim2* or *Syk*. In addition, the list contains mice with genetic deficiencies of the small GTPases, *Cdc42, Rhoa* or *Rac1*; deficiencies of protein (tyrosine) phosphatases *Dusp3, Mpig6b* (G6b-B), or *Ptprj* (CD148); deficiencies of other adhesive receptors, such as *Itga2* (integrin α_2_), *Itgb1* (integrin β_1_) or *Cd36* (GPIV); defects linked to altered PS exposure, i.e., *Ano1 (*TMEM16A), *Ano6* (TMEM16F)*, Capn1* (calpain-1) or *Kcnn4*. Other mice with single gene deficiencies came from an earlier undertaking to find novel proteins implicated in thrombosis and hemostasis (23), namely *Anxa1* (annexin 1), *Apoe* (plasma lipoprotein component), *Bnip2* (CBL2-interacting protein), *Dlg4* (scaffold protein), *Fpr2* (formyl peptide receptor), *Grm8* (glutamate receptor), *Ifnar1* (interferon receptor) or *Vps13a* (vacuolar sorting protein). In addition, a mutated mouse strain with a *Plcg2* gain-of-function (GOF, constitutive active PLCγ2) was incorporated (46). Given the ability of antibodies JAQ1 and INU1 to specifically cause platelet depletion from GPVI or CLEC2, respectively, after *in vivo* injection into mice (5) we also included strains with such antibody-induced GPVI and/or CLEC2 deficiencies.

**Figure 2:**
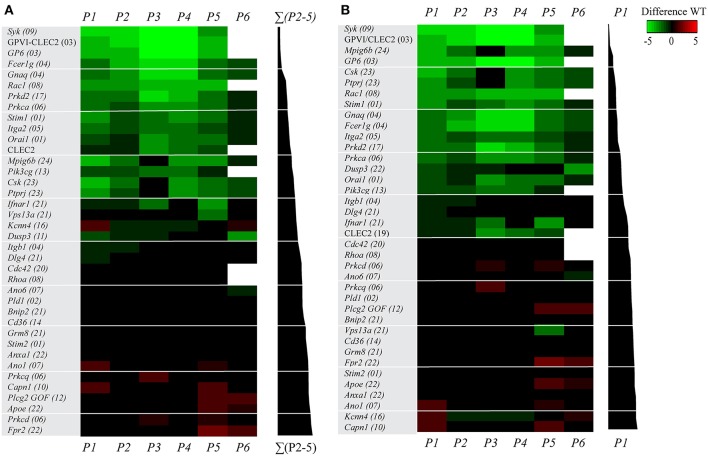
Multi-parameter comparison of collagen-dependent thrombus formation for 38 (genetically) deficient mice. Whole blood from mice with indicated genetic or antibody-induced defects as well as from corresponding wild-type mice was perfused over collagen-I. Parameters of thrombus formation were as indicated in Figure 1. For detailed information on mouse strains, see Table 1. Mean parameters per strain were scaled (0–10) across all wild-types (*n* = 22) and (genetically) modified mice (*n* = 38), as for Figure 1. Subtraction heatmaps showing differences between indicated genetic (or antibody-mediated) deficiency in comparison to wild-type, after filtering for differences outside the range of (composite) mean ± SD, to select relevant changes. **(A)** Ranking of genes based on effect on thrombus signature (Σ*P2-5*), as shown in black bars on the right. Colors indicate unchanged (black), decreased (green) or increased (red) parameters. **(B)** Ranking of genes based on effect on platelet adhesion (*P1*), as indicated in black bars. Colors represent unchanged (black), decreased (green) or increased (red) parameters. Non-subtracted heatmap values are provided in Supplementary Figure 2.

For assessment of the effects of (genetic) deficiency, scaled values per parameter were calculated for each of the 38 modified mouse strains and compared to those of the corresponding wild-types (Supplementary Figure 2). A subtraction heatmap was generated to pinpoint the effects of genetic modification, in which differences outside the range of (composite) means ± SD were considered as being relevant. The heatmap data could be ranked based on alterations in thrombus signature (Figure 2A) or on differences in platelet adhesion (Figure 2B). With either way of ranking, profound quantitative differences were observed in the majority of the thrombus formation parameters, when comparing the 38 modified mouse strains. Thrombus signatures were suppressed, in a decreasing order (Figure 2A), by deficiencies in *Syk*, GPVI/CLEC2, GPVI, *Fcer1g, Gnaq, Rac1, Prkd2, Prkca, Stim1, Itga2, Orai1*, CLEC2*, Mpig6b, Pik3cg, Csk*, and *Ptprj*. In contrast, this thrombus marker was elevated, in an increasing order, by deficiencies in *Prkcq, Capn1, Plcg2* (gain-of-function mutation), *Apoe, Prkcd*, and *Fpr2*.

The ranking based on altered platelet adhesion revealed several similarities, but also marked differences. Defects in *Syk*, GPVI*, Rac1, Stim1*, and *Itga2* resulted in a strong suppression of both platelet adhesion and thrombus signature. Relative larger effects on thrombus signature—in comparison to platelet adhesion—were apparent for mice with defects in *Fcer1g, Prkd2*, and *Prkca* (reduced thrombus formation), as well as for mice with defective members of the PKC family, *Prkcd* and *Prkcq* (increased thrombus formation). Relative increases in platelet adhesion were only seen for mice with deficiencies in *Kcnn4, Ano1*, and *Capn1*. In general, these heatmaps demonstrated that the ranking based on changes in thrombus signature (Figure 2A) can provide additional insight into the 'thrombogenic” consequences of a gene defect, in comparison to a ranking based on altered platelet adhesion (Figure 2B).

### Microfluidics Analysis of Thrombus Formation on Collagen and Other Surfaces

**Figure 3:**
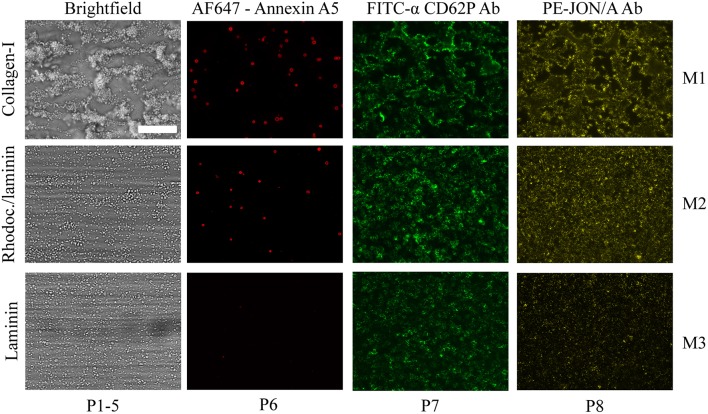
Microscopic imaging of whole blood thrombus formation on adjacent microspots. Whole blood from wild-type mice (DB 24) was flowed for 3.5 min at 1000 s^−1^ over three consecutive microspots of collagen-I (*M1*, upper row), rhodocytin/laminin/VWF-BP (*M2*, middle row), and laminin/VWF-BP (*M3*, lower row). Shown are representative brightfield and fluorescence microscopic images of the thrombi formed. Triple staining was performed with AF647-annexin A5, FITC-anti-CD62P Ab, and PE-JON/A Ab. Also indicated are the types of parameters (*P1-8*) taken from the image sets. Bar, 50 μm.

For a subset of 11 genetically modified mice strains, the same microfluidic device was used to assess whole blood thrombus formation on collagen-I (*M1*) plus two additional microspots, i.e., rhodocytin/laminin (*M2*) and laminin (*M3*), both co-coated with VWF-BP to induce shear-dependent platelet adhesion (Table 2). As clarified before, immobilized rhodocytin triggers CLEC2-induced platelet activation (26, 54), whereas laminin surfaces allow platelet adhesion via integrin α_6_β_1_ (22). For the same subset of mice, the formed thrombi were post-stained in three colors to quantify PS exposure (*P6*), P-selectin expression (*P7*) and integrin α_IIb_β_3_ activation (*P8*), using procedures previously established for human platelet thrombi (26). Representative images from each of the microspots using wild-type blood are depicted in Figure 3. In comparison to collagen-I (*M1*), rhodocytin/laminin (*M2*) was less thrombogenic, with only moderately activated platelets that formed small aggregates. The laminin microspot (*M3*) only triggered adhesion of a monolayer of spreading platelets.

**Figure 4:**
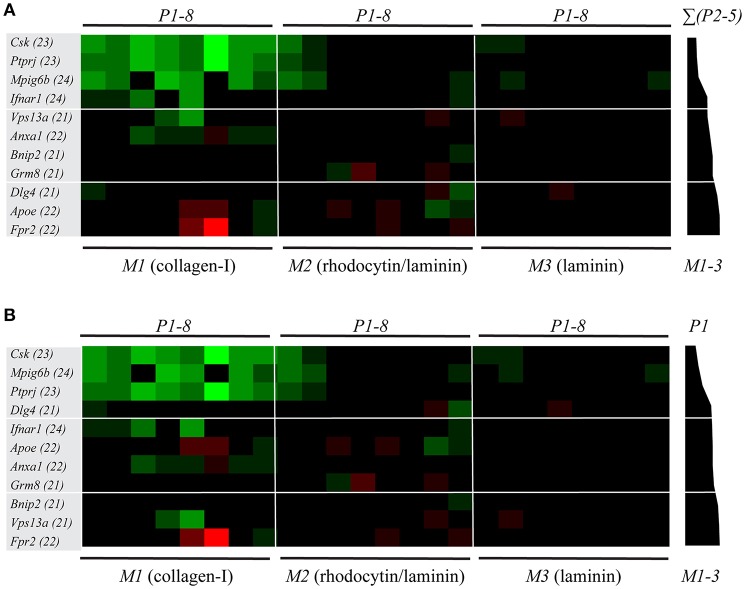
Multi-parameter comparison of thrombus formation on three microspots for 11 mouse strains with genetic deficiencies. Whole blood from mice with indicated genetic defects or corresponding wild-types was perfused over three microspots (*M1-3)*, as for Figure 3. Brightfield images (*P1-5*) and fluorescence images (*P6*, AF647-annexin A5; *P7*, FITC anti-CD62P Ab; *P8*, PE-JON/A Ab) were analyzed for each microspot. Per microspot, parameter values were univariate scaled (0-10) across all mouse strains. **(A)** Subtraction heatmap of differences between deficient and wild-type strains, filtered for changes outside the range of (composite) mean ± SD, to select relevant changes. Genes were ranked based on effects on thrombus signature (Σ*P2-5*) across microspots *M1-3*, as shown in black bars. Colors represent unchanged (black), decreased (green) or increased (red) parameters. **(B)** Subtraction heatmap with ranking based on gene effects on platelet adhesion (*P1*), as indicated in right black bars.

For these 11 genetically modified mouse strains plus corresponding wild-type mice, we again listed the scaled parameters (*P1-8*) for each microspot (*M1-3*) (Supplementary Figure 3). The ensuing subtraction heatmap revealed major reductions in platelet adhesion and thrombus signature at surface *M1* for the animals with deficiencies in *Csk, Ptprj, Mpig6b* (tyrosine protein kinase, phosphatases and ITIM receptor, respectively); and, to a clearly lesser extent, for deficiencies in *Ifnar1, Vps13a* and *Anxa1* (Figure 4). For the kinase and phosphatase knockouts, this extended to a reduction in platelet activation parameters (*P6-8*) at *M1*, and furthermore to lower *P1-2* values at the other microspots *M2-3*.

Markedly, for several of these mice, also gain-of-platelet-functions could be detected. In this case, increased parameters *P4-6* were distributed over *M1* (deficiencies in *Apoe* and *Fpr2*) and *M2* (deficiency in *Grm8*). Another remarkable finding was that, for the majority of mice, parameters at the laminin surface (*M3*) were unchanged. This suggested that laminin-platelet interactions are relatively insensitive to these genetic modifications. Next to the ranking based on changes in platelet adhesion (Figure 4B), the ranking of genes according to changes in overall thrombus signature (Figure 4A) appeared to be a valuable addition in the description of the alterations in platelet properties.

### Linking Microfluidics Outcomes to Thrombosis and Hemostasis *in vivo*

**Figure 5:**
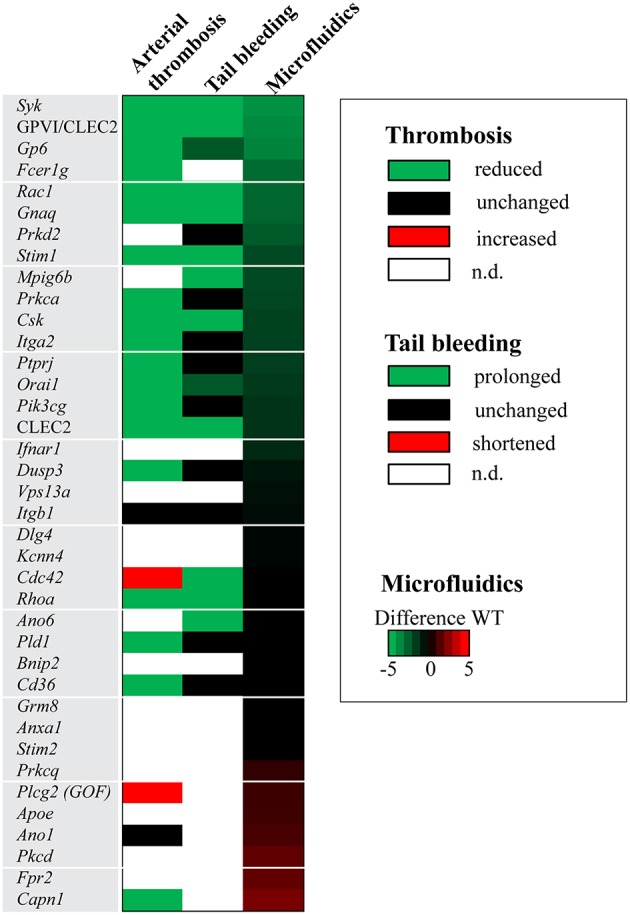
Effects of genetic modification on arterial thrombosis and tail bleeding in comparison to collagen-dependent thrombus formation using microfluidics. Effects of genetic or antibody-induced defects of concerning mouse strains on arterial thrombosis and tail bleeding was obtained from the literature (see Table 1). Effects were classified as being unchanged (black), antithrombotic/prolonged bleeding (green) or prothrombotic/shortened bleeding (red), according to procedures described before (23). White color indicates that information is lacking. Heat mapped data were ranked based on summative effects on all thrombus formation parameters (Σ*P1-5*) from surface *M1*.

Recently, we described a systematic procedure to compare the consequences of genetic knockout in mice for experimentally induced (collagen-dependent) arterial thrombosis and hemostasis (23). For the 38 deficiencies, it was thus of interest to compare the results of the current extended microfluidic assay (*M1, P1-5*) with the previously reported changes in arterial thrombosis tendency and tail bleeding *in vivo*. Hence, per modified mouse strain, the recorded changes (Table 1) were listed as being a reduced/unchanged/increased thrombosis phenotype and as a prolonged/unchanged/shortened bleeding time. Figure 5 gives the comparison of these *in vivo* effects with altered parameters of *in vitro* thrombus formation using microfluidics.

Strikingly, the majority of mice demonstrating an antithrombotic phenotype *in vivo* manifested with a larger or smaller reduction in collagen-dependent thrombus formation *in vitro* (Figure 5). We noted only a few exceptions: (i) mice deficient in *Cdc42* with an apparently prothrombotic phenotype, but for unknown reasons no effect *in vitro*; (ii) mice deficient in *Pld1* (where *in vitro* thrombus formation was only impaired at a higher shear rate of 1700 s^−1^) (14); and (iii) *Cd36*-deficient mice (requiring a thrombospondin surface for altered *in vitro* thrombus formation) (29). Also, the impaired arterial thrombosis reported for *Capn1*^−/−^ mice (27) did not match with a measured higher platelet adhesion under flow, although it should be noted that the latter mice showed a complex pattern of increased and decreased platelet activation parameters *in vitro* (28).

Markedly, in the mouse strains with a reduced arterial thrombosis tendency *in vivo*, only approximately half of these were accompanied with a hemostatic defect (Figure 5). This was also reflected by comparing *in vitro* thrombus formation (by microfluidics) with alterations in bleeding time. Mice with reduced *in vitr*o thrombus formation, but unchanged or slightly prolonged bleeding times, included animals with deficiencies in the collagen receptors *Gp6* and *Itga2*; the protein kinases *Prkd2* and *Prkca*; and the protein phosphatases *Ptprj* and *Dusp3*. This may suggest that the collagen receptors (and hence collagen itself) and the (de)phosphorylating proteins within the downstream signaling pathways are not essential for hemostasis. This may suggest that the collagen receptors (and hence collagen itself) and the (de)phosphorylating proteins are not uniquely—perhaps redundantly—required for platelet functions at lower shear rates such as during hemostasis. The same mouse genes were also previously characterized as having a distinct role in arterial thrombosis and hemostasis (23).

### Network Modeling to Predict Novel Proteins Implicated in Mouse Platelet Functions

**Figure 6:**
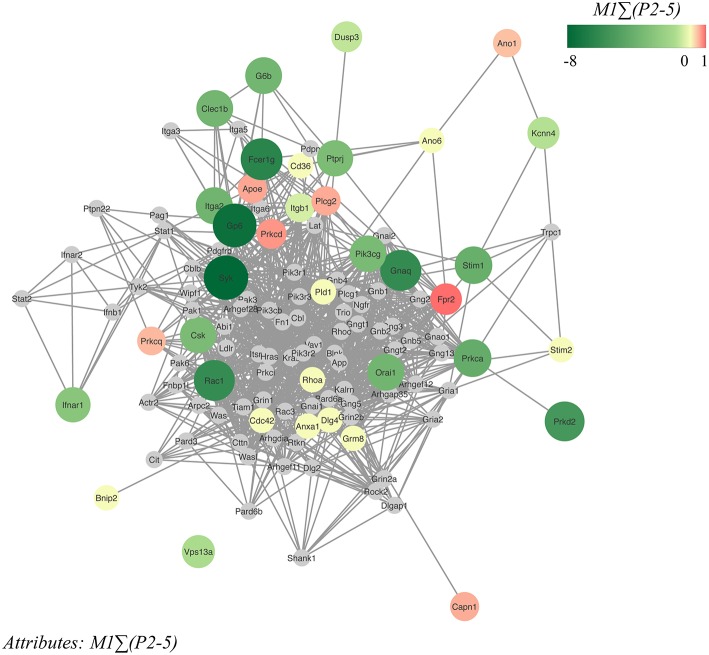
Network of protein-protein interactions in collagen-dependent thrombus formation. Network, constructed from the STRING database and visualized in Cytoscape, of murine protein-protein interactions with as seed the 37 investigated core genes. The network contained 117 nodes (37 core nodes, 80 novel nodes) and 1142 edges. Core nodes of the network were color- and size-coded based on altered thrombus signature (Σ*P2-5*) at surface *M1*. Green color intensity (larger size) of nodes points to a stronger reducing effect, and red color intensity (large size) to a stronger stimulating effect in comparison to wild-type. Novel nodes are indicated in gray.

Based on the 37 different mouse genes/proteins that were analyzed for effects on collagen-dependent whole blood thrombus formation, we constructed a network using the biological STRING (Search Tool for the Retrieval of Interacting Genes/Proteins) database, in order to be capable to depict additional protein-protein interactions. Accordingly, a network was established containing in total 117 nodes (37 core and 80 novel nodes) and 1142 edges (interaction score: medium to highest confidence: 0.40–0.99). Reactome pathways that were highly represented in the network were: platelet activation, signaling and aggregation (count in gene set: 37 of 242; false discovery rate: 3.25e^−39^), hemostasis (count in gene set: 44 of 489; false discovery rate: 1.5e^−38^), signal transduction (count in gene set: 68 of 2430; false discovery rate: 2.14e^−32^), GPVI-mediated activation cascade (count in gene set: 18 of 34; false discovery rate: 2.07e^−26^) and G alpha (12/13) signaling events (count in gene set: 18 of 68; false discovery rate: 2.64e^−22^). The 37 core nodes were color- and size-coded, based on the established gene effects on thrombus signature *M1P*Σ*(2-5)*, and then indicated three typical clusters of genes/proteins with large size effects (Figure 6): *(i) Gp6* with associated receptors *Fcer1g* and kinase *Syk* (cluster score: 7; #nodes: 19; #edges: 63); *(ii)* the low-molecular weight GTPases *Rac1* and *Cdc42* together with *Itga2* and *Itgb1* (cluster score: 5.24; #nodes: 22; #edges: 55); and *(iii)* Ca^2+^-regulating signaling components, *Stim1, Stim2* and *Orai1* (cluster score: 4; #nodes: 4; #edges: 6). Out of the 80 novel nodes, 12 genes have been previously shown to modify *in vivo* arterial thrombosis and/or bleeding (*Cblb, Cttn, Gnai2, Gria1, Itga6, Lat, Lcp2, Ldlr, Pik3cb, Pik3r1, Rock2, Vav1*) (23). Color-coding of the same network nodes for gene effects on platelet adhesion revealed as most notable changes the above-mentioned gain-of-function effects of *Ano1, Kcnn4 and Capn1* (Supplementary Figure 4).

## Discussion

In this paper, we applied standardized analysis procedures in order to allow detailed and quantitative comparison of the changes in platelet functions in multiple genetically modified mice, using microfluidic methods of collagen-dependent thrombus formation under shear conditions. For this purpose, we re-analyzed sets of microscopic brightfield and fluorescence images, using defined semi-automated scripts, resulting in quantitative parameters of platelet adhesion, platelet aggregation (defined as thrombus signature) (26), and platelet activation. The underlying rationale was that earlier captured images provide more relevant information than only a platelet surface area coverage, and thus can provide additional insight into the complex process of thrombus formation. For a subset of mice, it was also possible to extend this complex phenotyping of thrombus formation to other, non-collagen surfaces with additional parameters of platelet activation.

Recent work has shown that multiple platelet function assessment by whole blood microfluidic assays provides novel insights, for instance into the changes in human platelets linked to normal genetic variation (26, 62) and in mouse platelets due to co-activation by interacting chemokines (63). The present comparative quantitative analysis of changes in thrombus formation in multiple genetically or antibody-induced modified mice now also allows to evaluate the assay outcomes for changes in specific platelet functions. Here, we could identify a partial distinction between genes/protein affecting platelet adhesion and those altering platelet aggregation properties (collectively termed as thrombus signature). For instance, it seems that the murine *Csk, Mpig6b* and *Ptprj* have a relatively large role in flow-dependent platelet adhesion. Markedly, for *Csk, Mpig6b*, and *Ptprj* this effect is extended to also a reduced adhesion at the non-collagen surface *M2*. On the other hand, the processes of platelet adhesion and aggregate formation are also related, as shown by an overall consistent correlation between adhesion and aggregation parameters (Kendall's tau-b = 0.42-0.68). A similar conclusion was drawn earlier from the analysis of thrombus formation in blood samples from 94 healthy subjects, also pointing to the existence of a subject-dependent thrombus signature (26).

In comparison to the collagen surface (*M1)*, for the 11 mouse strains analyzed, we observed in general smaller gene effects at the two other surfaces (*M2-3*), which mediate platelet adhesion via GPIb-V-IX and α_6_β_1_ with or without CLEC2 (rhodocytin). Accordingly, it seems that, at least in part, distinct sets of genes/proteins are implicated in the platelet adhesion to non-collagen surfaces than to the collagen surface. Clearly *in vivo* thrombus formation is not purely mediated by collagen, but rather is the result of platelet interactions with a mixture of different extracellular matrix proteins.

The present data set also includes new findings with unpublished blood samples from mice deficient in *Rhoa* and *Syk*. The *Rhoa* defect did not appear to influence platelet adhesion nor thrombus signature parameters at the shear rate of 1000 s^−1^, which is in support of the earlier conclusion that platelet RHO-A becomes relevant for thrombus formation at high (pathological) shear rates (13). In contrast, genetic deficiency in *Syk* resulted in a strong reduction of all platelet and thrombus parameters on collagen. This highlights the importance of SYK signaling in collagen-dependent thrombus formation.

Given the earlier established correlation between the outcome of microfluidic tests (in terms of platelet surface area coverage) and experimental arterial thrombosis in mice (23), it was of interest to evaluate for the current mouse strains how a more detailed analysis (considering more thrombus parameters) contributes to this relationship. Such a comparison clearly has limitations, such as the wide variety of methods and test outcomes of the *in vivo* thrombosis measurements, making a clear differentiation between moderate and strong phenotypes difficult (23); and furthermore, the absence of coagulation and vessel wall components other than collagen in the *in vitro* approach. Nevertheless, a ranking of the investigated mouse strains according to overall changes in thrombus parameters (including platelet adhesion and aggregation) showed a good reflection with published changes in arterial thrombosis *in vivo*. On the other hand, for a considerable set of genes, a changed thrombus formation *in vitro* (and mostly *in vivo*) was not associated with an altered bleeding time. In several cases, a discrepancy is well explainable. For instance, defects in platelet *Clec1b* (as a non-collagen receptor) (64) or in *Ano6* (an isolated defect of platelet-dependent coagulation) (18), will not be picked up by flow assays over collagen with anticoagulated blood. A striking example is *Gp6*. Whereas, GPVI deficiency overall impairs thrombus formation *in vitro* as well as arterial thrombosis *in vivo*, mouse tail bleeding times are only moderately prolonged (37, 38). In line with that, mild bleeding symptoms have been reported in patients with a defect in the *GP6* gene (65, 66). Hence, this gene does likely have a restricted role, which is in line with the constructed network indicating multiple GPVI-linked proteins that contribute to both thrombosis and hemostasis.

Translation of the current evaluation of genes in murine thrombus formation to human pathophysiology can—taking into account the above—provide better insight into the genetic background of human platelet function abnormalities and how these can relate to thrombotic and bleeding disorders. Indeed, for homologs of several of the genes analyzed in this paper, e.g., human *ORAI1* and *STIM1*, mutations have been identified that link to defective collagen-dependent thrombus formation *in vitro* (67). By applying an extended image analysis, a detailed description of the formed thrombi can be generated. This can aid in the identification of the defective part (platelet adhesion, aggregation or procoagulant response) of the process of thrombus formation in patients with an unexplained increased risk for either thrombosis or bleeding. Extended analysis of the available mouse data and summation in the form of a network can also contribute to a more targeted approach to select novel candidate genes and proteins, possibly affecting platelet functions in a positive or negative way.

Moreover, by applying new knowledge and techniques, in the present paper, new insights were gained from previously performed mouse experiments. In such a manner, our study may contribute to the 3R approaches by reducing, refining and replacing animal experiments.

Taken together, we conclude that the presently developed multi-parameter analysis of thrombus formation on microspots using microfluidics can be used to: (i) determine the severity of platelet abnormalities; (ii) distinguish between altered platelet adhesion, aggregation and activation; and (iii) elucidate both collagen and non-collagen dependent platelet changes. This approach may thereby aid in the better understanding and better assessment of the changes in platelets that affect arterial thrombosis and hemostasis.

## Data Availability

The raw data supporting the conclusions of this manuscript will be made available by the authors, without undue reservation, to any qualified researcher.

## Ethics Statement

For original data, experiments were approved by the district government of Lower Franconia (Bezirksregierung Unterfranken) and by the Animal Experimental Committee in Hinxton/Cambridge. For previously published data, experiments were approved by the local Animal Experimental Committees as indicated in the original publications (see references in Table 1).

## Author Contributions

MN and JvG analyzed and interpreted the data and wrote the manuscript. SdW and MK analyzed the data. RV created scripts used for image analysis. DA, AB, ME, KK, CO, JP, IP, AP, JM, MG, YS, and CW provided the mice, other tools, and revised the manuscript. DS and BN provided the mice, original published and unpublished images, and revised the manuscript. JH and CB provided expert supervision, analyzed and interpreted data, and wrote the manuscript.

### Conflict of Interest Statement

JH is a co-founder and shareholder of FlowChamber. The remaining authors declare that the research was conducted in the absence of any commercial or financial relationships that could be construed as a potential conflict of interest
