# Supplementary material for: Comparative Analysis of Microfluidics Thrombus Formation in Multiple Genetically Modified Mice: Link to Thrombosis and Hemostasis
Source: Front Cardiovasc Med. 2019 Jul 30;6:99. doi: 10.3389/fcvm.2019.00099 (PMC6682619; doi:10.3389/fcvm.2019.00099)
Supplement: Supplementary file 1 [file Data_Sheet_1.PDF]

Supplementary Material

Supplementary Figures

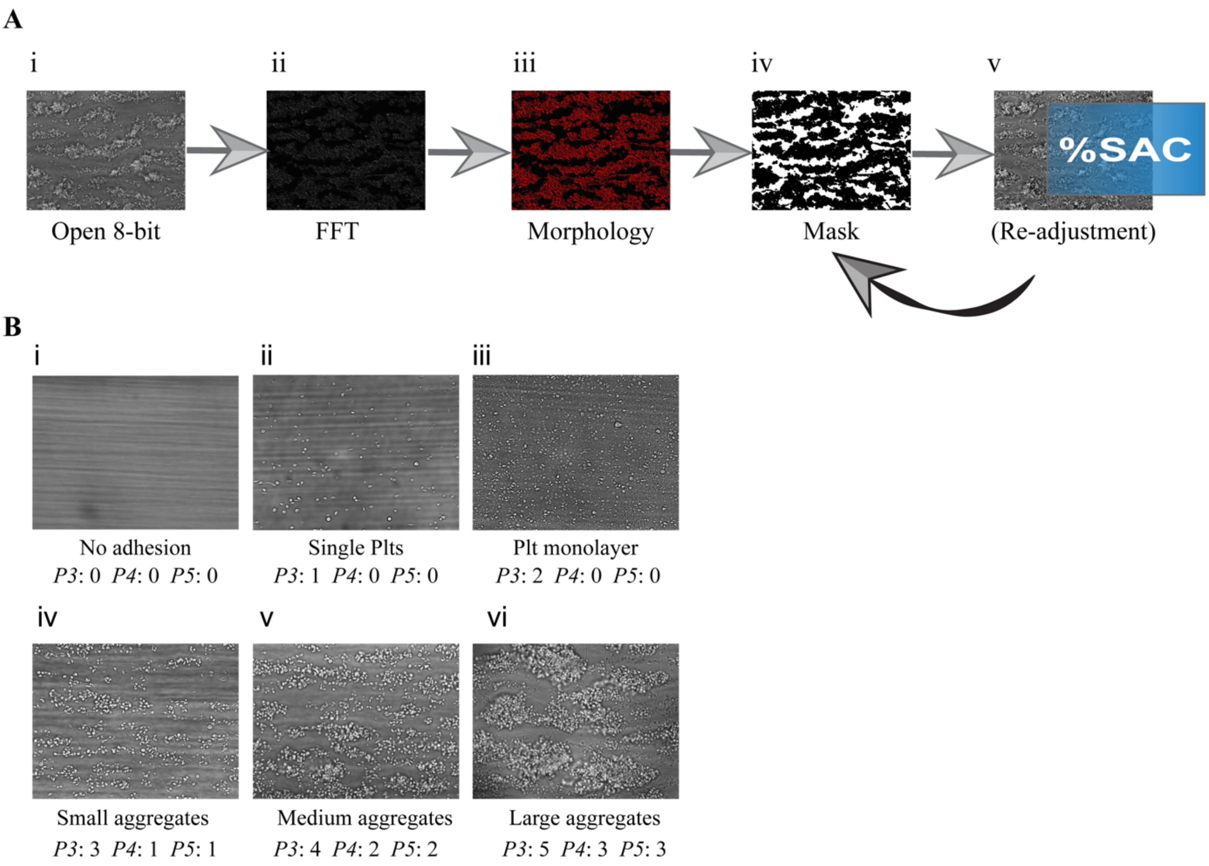

Supplementary Figure 1. Flow-chart of image analysis procedures. A) Brightfield and fluorescence microscopic images were analyzed for surface-area coverage (SAC%) using scripts written in Fiji. Separate analysis steps were: *(i)* opened image in 8-bit; *(ii)* fast Fourier transformation (FFT) to correct for background noise; *(iii)* morphology filter with vertical/horizontal dilate and erode steps to enhance relevant structures; *(iv)* thresholding and conversion to mask overlays; *(v)* back-loop of manual re-adjustment of threshold, if not adequately set. Different scripts were used per image type and fluorescent label. **B**) Reference brightfield images for scoring of parameters *P3* (thrombus morphology score, scale 0-5), *P4* (thrombus multilayer score, scale 0-3), and *P5* (thrombus contraction score, scale 0-3).

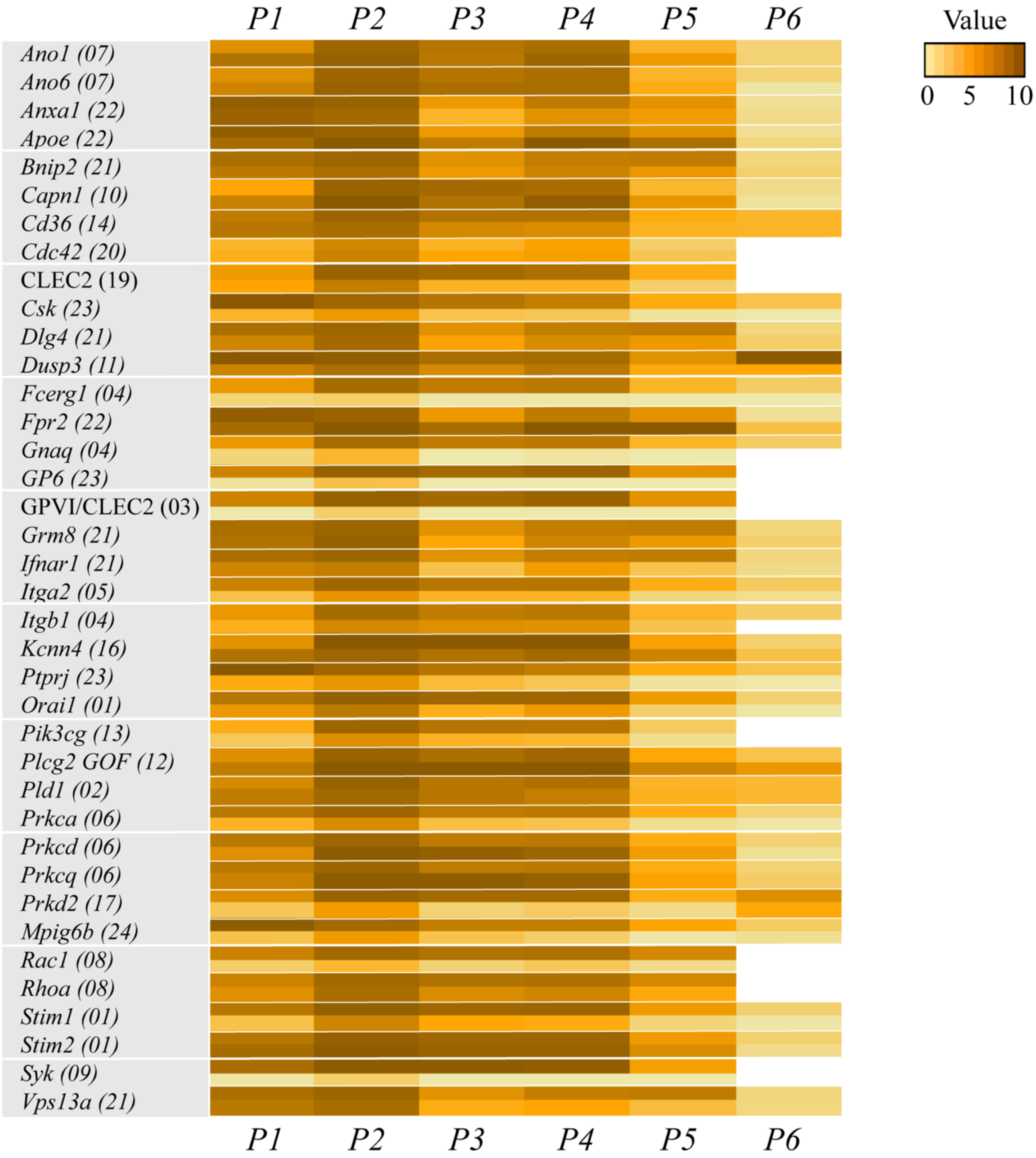

Supplementary Figure 2. Overall effect of genetic modification or antibody treatment on parameters of collagen-dependent thrombus formation per gene and parameter. Blood from 22 corresponding wild-type (upper rows) and 38 modified (lower rows) mouse strains (databases as indicated in brackets) was perfused over collagen at shear rate of 1000 s^{-1} (in two cases 1700 s^{-1}). For full details, see Table 1. Heatmap is shown of mean parameters, after scaling (0-10) across all mouse strains. Parameters: *platelet adhesion*: *P1* (platelet SAC%); *thrombus signature: P2* (platelet aggregate SAC%), *P3* (thrombus morphology score), *P4* (thrombus multilayer score), *P5* (thrombus contraction score); and *platelet activation*: *P6* (phosphatidylserine exposure).

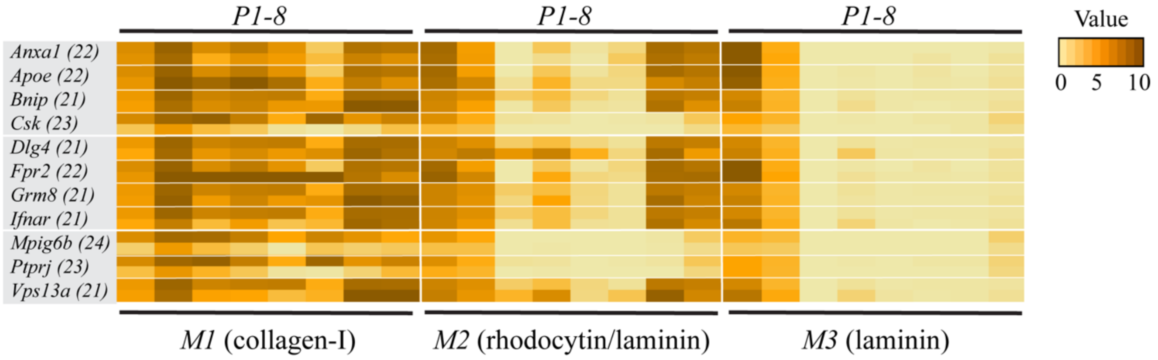

Supplementary Figure 3. Multi-parameter comparison of thrombus formation on three microspots for 11 genetically modified mouse strains. Blood from corresponding wild-type (upper rows) and 11 modified (lower rows) mouse strains (databases as indicated in brackets) was perfused over microspots *M1-3* at shear rate of 1000 s^{-1}. Mouse strains were arranged based on alphabetical order. For full details, see Table 1. Heatmap of mean parameters, scaled (0-10) across all mouse strains.

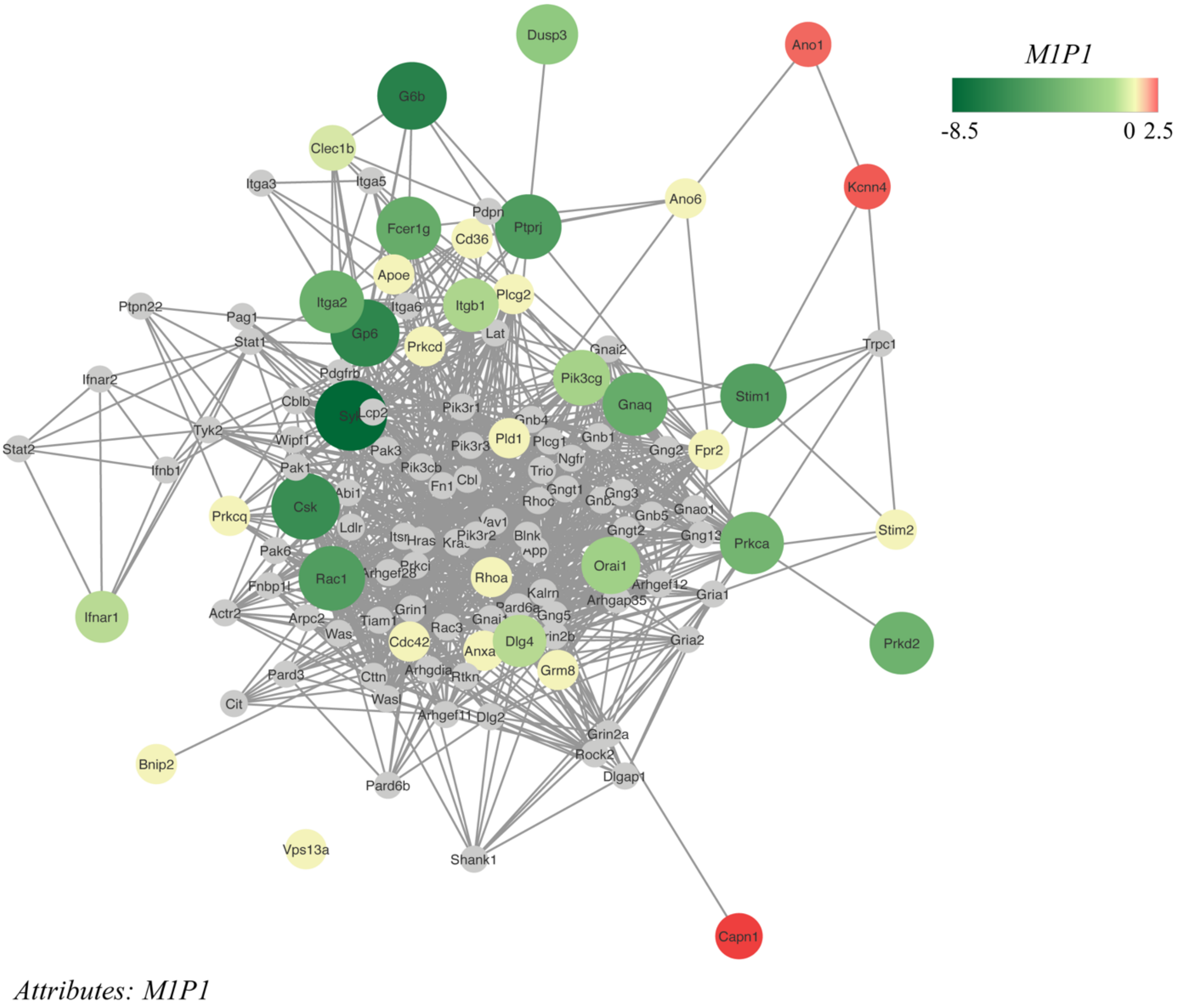

Supplementary Figure 4. Network of protein-protein interactions in collagen-dependent thrombus formation: platelet adhesion attributes. Network, constructed as in Figure 6, but with the core nodes color- and size-coded, based on altered platelet adhesion (*P1*) at surface *M1*.

Supplementary Table

Parameter	*CV%*
Platelet adhesion	
P1, platelet surface area coverage	23.01
Thrombus signature	
P2, platelet aggregate coverage	24.88
P3, thrombus morphology score	3.40
P4, thrombus multilayer score	8.19
P5, thrombus contraction score	11.84

Supplementary Table 1. Mean coefficient of variation (CV%, SD/mean) of parameters *P1-5* for collagen-dependent thrombus formation, comparing the results from 22 wild-type mice dataset (for databases, see Figure 1).
